# Bioactive Peptides and Proteins from Centipede Venoms

**DOI:** 10.3390/molecules27144423

**Published:** 2022-07-11

**Authors:** Yalan Han, Peter Muiruri Kamau, Ren Lai, Lei Luo

**Affiliations:** 1 Key Laboratory of Animal Models and Human Disease Mechanisms of the Chinese Academy of Sciences/Key Laboratory of Bioactive Peptides of Yunnan Province, KIZ-CUHK Joint Laboratory of Bioresources and Molecular Research in Common Diseases, National Resource Center for Non-Human Primates, Kunming Primate Research Center, National Research Facility for Phenotypic & Genetic Analysis of Model Animals (Primate Facility), Sino-African Joint Research Center, and Engineering Laboratory of Peptides, Kunming Institute of Zoology, Kunming 650107, China; hanyalan@mail.kiz.ac.cn (Y.H.); peter@mail.kiz.ac.cn (P.M.K.); 2 University of Chinese Academy of Sciences, Beijing 100049, China; 3 Center for Evolution and Conservation Biology, Southern Marine Science and Engineering Guangdong Laboratory (Guangzhou), Guangzhou 511458, China

**Keywords:** venom, centipede, peptide, protein, drug development

## Abstract

Venoms are a complex cocktail of biologically active molecules, including peptides, proteins, polyamide, and enzymes widely produced by venomous organisms. Through long-term evolution, venomous animals have evolved highly specific and diversified peptides and proteins targeting key physiological elements, including the nervous, blood, and muscular systems. Centipedes are typical venomous arthropods that rely on their toxins primarily for predation and defense. Although centipede bites are frequently reported, the composition and effect of centipede venoms are far from known. With the development of molecular biology and structural biology, the research on centipede venoms, especially peptides and proteins, has been deepened. Therefore, we summarize partial progress on the exploration of the bioactive peptides and proteins in centipede venoms and their potential value in pharmacological research and new drug development.

## 1. Introduction

‘Struggle for survival’, a linchpin of Darwinian evolution theory, is an incessant natural phenomenon where all living organisms must adapt to diverse environmental factors, cope with competition, and ultimately win in natural selection [1]. In natural ecosystems, organisms with advantageous mutations or traits are conferred better survival abilities. Such abilities might be due to environmental pressure, climatic changes, or extreme competition, where organisms that can withstand such challenges are selected for survival. One such important consequence of natural selection is venom production from single-cell protozoans to metazoan primates [2]. This phenomenon, whereby distantly related organisms adapt to similar requirements, can be referred to as convergent evolution. For example, centipedes envenomate their enemies or prey as a defensive and offensive strategy.

In the animal kingdom, venomous organisms are represented in a broad range of phyla, both invertebrates and vertebrates occupying different ecological habitats [2,3,4,5]. Venom production marks a vital adaptation and survival strategy in natural ecosystems riddled with strenuous competition for limited resources [2]. Venom comprises a complex cocktail of active pharmacological peptides and/or proteins produced by an animal’s specialized venom system [6,7]. Such peptides serve defensive or predatory roles through a range of actions, including killing cells (cytotoxins, necrotoxins), targeting and damaging muscles (myotoxins), and impairing the nervous system (neurotoxins).

Centipedes are terrestrial, predatory arthropods belonging to the phylum Arthropoda, subphylum Myriapoda, and class Chilopoda. As one of the four major lineages of the myriapod, centipedes include 3300 to 3500 species belonging to five extant orders, namely Scutigeromorpha, Lithobiomorpha, Craterostigmomorpha, Geophilomorpha, and Scolopendromorpha, and an extinct fossil order, Devonobiomorpha [8,9]. They are present on every continent except Antarctica, with the greatest diversity occurring in the tropics and warm temperate regions [10]. Most centipedes live in rotten leaves and soil in wooded areas or under stones, bark, or wood, although some live in grasslands, deserts, caves, and coastal regions [10].

Centipedes are excellent predators. Although centipede bites rarely cause death in humans, it is fatal to insects and crustaceans. Similar to many spider toxins, centipede venoms show good insecticidal activity [11]. Four neurotoxins, μ-SLPTX-Ssm1a, κ-SLPTXSsm1a, 2a and 3a, isolated and characterized from *Scolopendra paucifera*, have apparent insecticidal toxicity to blowfly larvae, adult blowflies, cockroaches, and mealworms [12]. Centipedes can kill and feed on vertebrates, such as bats, rats, amphibians, and reptiles [13,14]. In addition, the toxicity varies among centipedes. For instance, mice’s median lethal dose (LD_50_) values were 0.16 and 0.012 g/kg for *Scolopocryptops ferrugineus* and *Otostigmus scabricauda*, respectively, a difference of more than 10-fold [8]. Although human death from centipede bites is rare, they can still induce severe complications, such as severe pain, swelling, hemorrhage, tissue necrosis, nausea, vomiting, general rash, myocardial ischemia, and infarction [10,15]. These pathological symptoms induced by centipede envenomation indicate that centipede venoms are rich in diverse bioactive components acting on several systems.

In the last 30 years, with the highly developed methods of venom research, including cDNA library construction, protein sequencing, mass spectrometry, genomic analysis, transcriptome analysis, proteome analysis, and structural biology techniques, venoms from many animals have been intensively studied [15,16]. Recently, a new venom-peptide family named HAND toxins and a recombinant toxin named Cryptoxin-1 from centipedes were successively identified [17,18]. Centipede venoms have recruited gene families by horizontal gene transfer between bacteria, fungi, and oomycetes [19]. Centipede venom composition between male and female have abundance differences that show significant sex-based variation [20]. These studies presented discoveries about biochemical characteristics and novel centipede venom genetic evolution mechanisms. The in-depth exploration of toxins is not only beneficial to understanding the survival strategies of venomous animals but also helpful in screening the leading molecules with potential therapeutic uses. Here, we describe the peptides/proteins from centipede venoms with pharmacological activities targeting the nervous, blood, and immune systems. The representative centipede venom components noted herein are summarized in Table 1.

## 2. Centipede Toxins Acting on the Nervous System

Centipedes are excellent predatory arthropods. They deploy a broad set of bioactive peptides to capture prey or defend against predators [23,38,47,48,49,50]. Neurotoxins are the primary predation and defense peptides in centipede venom and also important ingredients that have made significant progress in revealing the biological activities and action mechanisms in recent research. These components act on a wide array of targets, mostly the ion channels, either by activating or inhibiting their electric activity.

### 2.1. Toxins Targeting Voltage-Gated Sodium Channels

**Figure 1:**
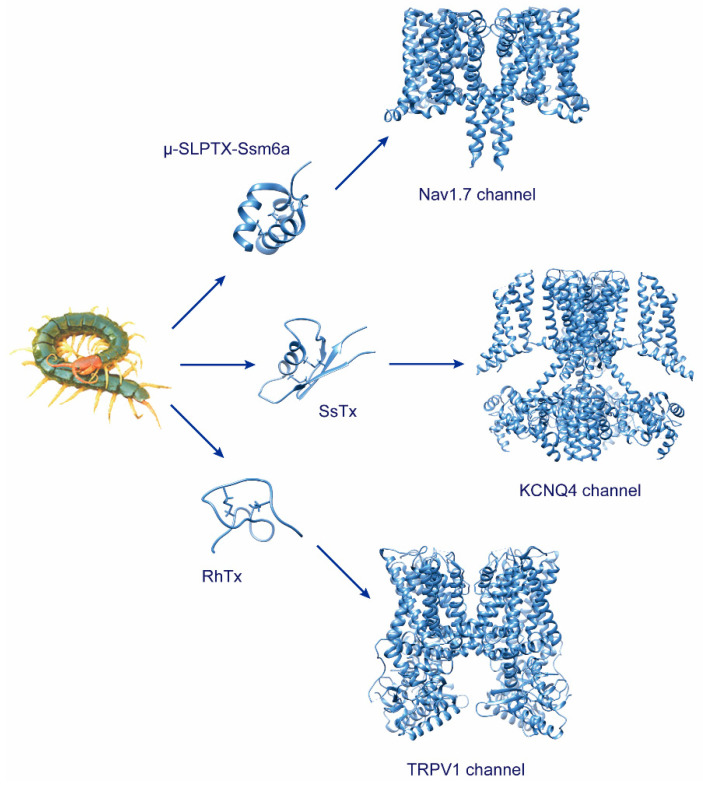
Representative centipede toxins and related ion channel.

Voltage-gated sodium channels (Na_V_) are critical molecular determinants of electrical impulses (action potentials) initiation and propagation, which underlie the electrical hyperexcitability characteristic of chronic inflammatory and neuropathic pain [51,52]. We have made in-depth research on the venom of the Chinese red-head centipede, *Scolopendra subspinipes mutilans* L. Koch. μ-SLPTX-Ssm1a was a selective TTX-sensitive (TTX-S) Na_V_ channel inhibitor with the complete amino acid sequence ADNKFENSLRREIACGQCRDKVKCDPYFYHCG [12]. Interestingly, another selective Na_V_ channel inhibitor with an almost identical N-terminal sequence of μ-SLPTX-Ssm1a was further discovered from the *S. subspinipes mutilans*. μ-SLPTX-Ssm6a consists of 46 amino acid residues, yielding a molecular mass of 5318.4 Da. By using whole-cell patch-clamp recordings, this peptide potently inhibited the Na_V_1.7 channel with a half-maximal inhibitory concentration (IC_50_) of ~25 nM, which showed a much higher selective than other human sodium channels subtypes (Figure 1). In addition, μ-SLPTX-Ssm6a exhibited an analgesic effect than morphine in formalin-induced pain models. Moreover, μ-SLPTX-Ssm6a showed an almost equal analgesic effect with morphine in thermal and acid-related pain models [21]. Many neurotoxins from venomous animals such as scorpion, spider and snail also target Na_V_1.7. For instance, ProTx-II, a tarantula toxin, selectively targets the Na_V_1.7 channel, yielding an IC_50_ of 0.3 nM [53]. Similar to the effect of μ-SLPTX-Ssm6a on rat DRG neurons, ProTx-II shifted the conductance–voltage relationship in a depolarizing direction [53] despite their different structures. μ-SLPTX-Ssm6a is composed of three α helix structures, while ProTx-I contains two anti-parallel β-folds, which belong to the inhibitory cystine knot family [54].

By the venomic and transcriptomic analysis of centipede *Scolopendra subspinipes dehaani*, Liu et al., identified only one group of peptides with five members coding for an identical mature peptide [22]. The Mexican centipede *Scolopendra viridis* crude venom was also reported to weakly inhibit hNa_V_1.2 and hNa_V_1.6 channel subtypes, indicating the existence of sodium channel inhibitors in *S. viridis* [55]. With the help of peptidomics combined with the cDNA library, we uncovered another precursor that has activity on the sodium channel [56].

### 2.2. Toxins Targeting Voltage-Gated Potassium Channels

Voltage-gated potassium channels (K_V_) distinctively modulate firing action potentials with the Na_V_ channel. The Na_V_ channel depolarizes the membrane potential while the K_V_ channel repolarizes the membrane potential. The K_V_ modulators account for a considerable portion in centipede venom. For instance, 10 families of K_V_ inhibitors were identified from the *S. subspinipes dehaani* [22]. Moreover, the selectivity and potency of K_V_ modulators are variable. SSD559, the most potent K_V_ inhibitor, dose-dependently inhibits potassium channels in DRG neurons, and the IC_50_ for potassium channel inhibition was 10 nM [22]. In contrast, κ-SLPTX-Ssm3a was a weaker K_V_ inhibitor. Application of 200 nM κ-SLPTX-Ssm3a on K_V_ channels of dorsal root ganglion (DRG) neurons inhibits 25 ± 5% currents, and κ-SLPTX-Ssm3a does not entirely diminish the potassium peak currents even up to 5 µM, indicating that κ-SLPTX-Ssm3a is a weak inhibitor of potassium channel [12].

Based on centipede toxicity tracking, we isolated Ssm spooky toxin, SsTx, from the *S. subspinipes mutilans*. The structure of SsTx is polarized, with basic amino acids of arginine (position 12) and lysine (position 13) forming a positively charged surface. Further analyses showed that SsTx potently inhibited the KCNQ family with R12 and K13 on SsTx, and formed two pairs of salt bonds with residues 288 (aspartic acid) and 266 (aspartic acid) on KCNQ4, respectively (Figure 1). In addition, SsTx potently disrupts the cardiovascular, nervous, respiratory and muscular systems in rodent and mammal models [15]. In a further study, we showed that SsTx also inhibits the K_V_1.3 channel, amplifying the broad-spectrum destructive effect by inhibiting the KCNQ family, and shows that SsTx plays a key role in centipede defense and predation [57]. Yajamana et al. reported that SsTx, alone with three identified peptides (SsdTx1-3), could also inhibit the pore of the human Kir6.2 channel [58]. Another analog of SsTx, SsTx-4, effectively inhibits Kir1.1, Kir4.1, and Kir6.2/SUR1 channels, which are candidate targets for treating hypertension, depression, and diabetes, respectively [23]. Similar peptides were also discovered from other venomous species, including cone snails. κ-, κA-, κM- and I- superfamilies of conotoxins were reported to inhibit K_V_ channels by interacting with the voltage-sensing or pore domains [59]. In comparison, most of the K_V_ channel modulators from the centipede venoms target the pore region.

### 2.3. Toxins Targeting Voltage-Gated Calcium Channels

Both activators and inhibitors of the Ca_V_ channel have been discovered in centipede venoms. We found that ω-SLPTX-Ssm1a potently activated voltage-gated calcium channel (Ca_V_) in rat DRG neurons. Functionally, 10 µM ω-SLPTX-Ssm1a increased the calcium channel currents by ~120%, while ω-SLPTX-Ssm2a inhibits calcium channels in a dose-dependent manner. Functionally, 500 nM and 2.5 μM ω-SLPTX-Ssm2a inhibited the calcium channel current’s amplitude by 45% and 80%, respectively, yielding an IC_50_ of about 1590 nM [12]. SSD1052, a calcium channel inhibitor, was isolated from *S. subspinipes dehaani* crude venom. Ten nanometers of SSD1052 reversibly blocks the Ca_V_ current amplitude by 8.6% [22]. To date, most Ca_V_ modulators from centipedes are antagonists, and ω-SLPTX-Ssm1a is the only agonist. These peptides are structurally diverse with variable disulfide bonds, and all possess similar molecular mass (about 6 kDa). Ca_V_ modulators from other venomous animals, such as cone snails, have been extensively studied. Representative conotoxins, ω-GVIA and ω-MVIIA, potently inhibit N-type calcium channels. ω-MVIIA has been approved by the U.S. Food and Drug Administration to treat chronic pain. Thus, further detailed investigation of the pharmacological properties of centipede venoms is essential.

### 2.4. Toxins Targeting TRPV1 Channel

As we exhibited earlier, centipede toxins are rich in neurotoxins. The Transient Receptor Potential Vanilloid 1 (TRPV1) channel mediates the heat and pain sensation in the periphery nervous system [60]. Yang et al. reported the discovery of a compact toxin from *S. subspinipes mutilans*. The gene encoding this toxin translated into a 69 aa, which yielded a toxin with 27 amino acids after post-translation modification. RhTx binds tightly to the charge-rich outer pore region of TRPV1 to induce severe pain and provides crucial structural information on the channel’s heat activation machinery (Figure 1) [24]. In addition, RhTx was used as a probe to investigate the heat-induced desensitization mechanism of the TRPV1 channel [61]. RhTx2 is an analog of RhTx with four more amino acids at the N-terminal. Functionally, RhTx2 desensitized the TRPV1 channel upon application to the extracellular domain, indicating that RhTx2 is also a good tool for the investigation of TRPV1 desensitization and a promising candidate for the development of new analgesics [25].

## 3. Centipede Toxins Acting on the Immune System

The immune response involves a complex myriad of biological processes that respond and protect against foreign factors. Several venom peptides attack the immune system, and as a defensive mechanism, the immune system is activated to recognize and counter their effect. One of the main responses against venom components is the release of anti-inflammatory agents to counterbalance toxin-induced inflammation [62]. Inflammation involves a series of immune responses, including inflammatory cytokine release, vascular changes, and recruitment of immune cells (dendritic cells, mast cells, neutrophils, and eosinophils). Until recently, the immune-related components from centipede venoms are rarely studied, except for 5-hydroxytryptamine and histamine [41,63]. Through *N*-terminal sequencing, allergen-related proteic venom components were identified from *Scolopendra viridicornis nigra* and *Scolopendra angulate* [64]. With the improvement of sequencing and mass spectrometry platforms, various antimicrobial peptides and anti-inflammatory peptides were discovered from the venom of the centipede.

Antimicrobial peptides (AMPs) derived from venoms have proven clinical efficacy in combating multidrug-resistant pathogens. Amphibians and insects are believed to be good resources for developing AMPs. Arthropods, especially centipedes, are also rich in various bioactive peptides. Scolopendrin I, the first antimicrobial peptide from a centipede, had no hemolytic or agglutination activity at concentrations lower than 30 µM [26]. Scolopin 1 and scolopin 2, with molecular masses of 2593.9 and 3017.6 Da, respectively, were also purified and characterized from the *S. subspinipes mutilans*. Both scolopin 1 and 2 exhibited potent antimicrobial activities against Gram-positive and Gram-negative bacteria and fungi, with moderate hemolytic activity [27]. LBLP, a lactoferricin B-like peptide from the whole bodies of adult centipedes, *S. s. mutilans*, shows potent antifungal activity. The antifungal mechanism revealed that LBLP changes membrane permeabilization by forming pores in the membrane with radii between 0.74 and 1.4 nm [28]. Other AMPs, such as scolopendin 1 and 2, were discovered from the whole centipede *S. subspinipes mutilans* by RNA sequencing [29,30]. Similar to the general antibacterial mechanism of most AMPs, scolopendin 2 forms pores in the microbial plasma membrane, then releases the cytoplasmic matrix, depolarizes the membrane potential, and eventually leads to microbial death [30]. The scolopendrasin I, II, V and VII peptides from the whole *S. subspinipes mutilans*, displayed antimicrobial and anticancer activities, of which scolopendrasin V exerted antimicrobial activities by binding the surface of the microbial cell membrane [31,32,33,34]. With the emergence of novel AMPs, more investigations on antibacterial mechanisms are in progress.

The whole-body extracts of centipede were reported to exert anti-inflammatory activities in rheumatoid arthritis and antitumor and immunostimulant [43,65]. Scolopendrasin IX, an antimicrobial peptide from *S. subspinipes mutilans*, targets the formyl peptide receptor 2 and mediates neutrophil activation. Functionally, scolopendrasin IX controls rheumatoid arthritis by inhibiting inflammatory cytokine synthesis [35].

## 4. Centipede Toxins Acting on the Blood System

An efficient blood circulation system, including in humans, is paramount for vertebrates’ survival. It serves as a key biological process for the survival of living organisms, such as transporting nutrients and oxygen to tissues and removing waste products. It also plays an essential defense role by transporting immune cells to target sites. Thus, a homeostatic balance is necessary for the general well-being of the organism. The coagulation cascade comprising key processes such as platelet aggregation, vasoconstriction, coagulation, and fibrinolysis activated immediately upon injury is a vital component that maintains the integrity of the immune system and maintains a hemostatic balance between clotting and excessive loss of blood. Through years of co-evolution, several animals have evolved elaborate strategies targeting and impairing the hemostatic system. For example, hematophagous organisms such as vampire bats, ticks, leeches, and mosquitoes inject saliva rich in peptides to prevent blood coagulation and ensure a continuous blood supply [66,67]. Although centipedes are not blood-feeding arthropods, proteic venom components that affect the blood system have also been found in centipede venom. For instance, the venoms of both centipede species, *Scolopendra viridicornis* and *Otostigmus pradoi*, had hemolytic activity on human erythrocytes [44].

Factor Xa (FXa) is essential in both extrinsic and intrinsic pathways for blood coagulation, which is also a candidate target for exploring and developing anti-thrombotic drugs [68]. TNGYT, a mature peptide with five amino acids, dose-dependently inhibited FXa, yielding an IC_50_ of 41.14 mg/mL. The TNGYT prolonged the activated partial thromboplastin time (aPTT) and prothrombin time (PT) both in vivo and in vitro [36]. Another short peptide SQL (Ser-Gln-Leu) was also isolated from the whole centipede *S. subspinipes mutilans*. SQL potently prolonged the aPTT and inhibited platelet aggregation [45]. Centipede acidic protein showed significant anti-atherogenic effects and improved hemorheological disorders and histopathological changes in rats fed an atherosclerotic diet [69]. Scolonase was isolated and characterized from the whole-body extract of *S. subspinipes mutilans*. As a serine peptidase, scolonase showed potent fibrinolytic activity, which converted Glu-plasminogen to plasmin by specific cleavage of the peptide bond Arg(561)-Val(562) [46].

## 5. Centipede Toxins Acting on Other Systems

Mohamed et al. reported for the first time that the enzymatic activity is present in *Scolopendra morsitans* venom [41]. Other enzymes, such as metallopeptidases, serine peptidases, γ-Glutamyl transpeptidase and phospholipase A2, are also rich in centipede venoms. Although centipedes could use their mandibles to help chew solid food before swallowing [38,50], these abundant enzymes may also favor extra-oral digestion of prey.

### 5.1. Metallopeptidases

By employing activity tests and sequence analysis, various studies have revealed that centipede venoms are rich in metallopeptidases [8,44,70,71]. The astacin-like metallopeptidases accounted for approximately 10% of all proteins in *Thereuopoda longicornis*. The astacin-like family of metallopeptidases is widely recruited in many animals, including cnidarians, cephalopods, hymenopterans, ticks, spiders, reptiles, and platypus [37,72,73,74,75,76,77]. With the improvement of transcriptomics and venom proteomics, four metallopeptidases were identified from the *Scolopendra viridis*, which all showed high sequence identity with the astacin-like family of metallopeptidases (M12A family) [70]. Likewise, adamalysin-like metallopeptidases were identified from the centipede Scolopocryptops sexspinosus, supporting the convergent recruitment of venom proteins [71]. To date, no putative metallopeptidase has been found in the *Scolopendra subspinipes dehaani* and *S. subspinipes mutilans*, possibly due to limitations of the analytical method employed. Jenner and Undheim proposed that some toxin families could be lost from centipede venoms during evolution by comparative proteo-transcriptomic analyses, which could explain the deficiency of metalloproteases [78].

### 5.2. Phospholipase A2

Phospholipase A2 (PLA2) enzymes are 13–14 kDa polypeptides composed of 115–133 amino acid residues conserved to 15 cysteine residues stabilized by 7-disulfide bridges [50]. PLA2s have been characterized by several organisms, including insects, arachnids, and reptiles. They induce various pathologies such as neurotoxicity, hemotoxicity, cardiotoxicity, inhibition of platelet function and anticoagulant activities [79]. In centipedes, PLA2s activity was reported in *S. viridis*, *S. subspinipes dehaani, S. viridicornis, and O. pradoi* [22,44,80]. Unlike other invertebrates or vertebrate PLA2s, centipede PLA2s are unique and form a sister-clade to Group X-related PLA2 [37,79]. Low PLA2s activity was detected in venoms of *S. viridicornis* and *O. pradoi* [44]. PLA2s are ubiquitous in many venomous snakes such as elapid, rattlesnake and pit viper. PLA2s isolated from elapid venom could hydrolyze phospholipids, whereas those isolated from viper venoms could not unless exogenous phospholipids were added [81,82,83]. In addition, PLA2s from elapid and viper were reported to exert hemolytic activities, while PLA2s from centipede exhibited no directly cytotoxic effects [22,82].

PLA2s are thought to hydrolyze glycerophospholipids and release lysophospholipids and fatty acids. However, the catalytic reaction was often removed from snake and toxic centipede venoms, which might explain the low phospholipase A2 activity of *S. viridicornis* and *O. pradoi* venoms. In addition, the phylogenetic analyses revealed that centipede PLA2 shares a higher sequence identity with snake phospholipases than insects or arachnids [22].

### 5.3. γ-Glutamyl Transpeptidase

γ-Glutamyl transpeptidases (GGTs) are reported to regulate primarily oxidative stress responses and xenobiotic detoxification [84]. By utilizing transcriptome analysis, GGTs were found to be abundant in the venoms of almost all tested species, which were lowly distributed in the venoms of *E. rubripes, T. longicornis,* while highly expressed in the venoms of *S. alternans, S. morsitans, and C. westwoodi* [37], indicating that GGT might be a crucial component of centipede venom. Liu et al. reported the presence of GGT (SSD14) in the *S. subspinipes dehaani* using venomic and transcriptomic analysis. SSD14 dose-dependently induced human platelet aggregation and hemolysis of red blood cells from rabbits and mice [22]. However, the primary function of GGT does not seem to target the vertebrate hemostasis since the body size of the centipede is relatively small, and there are not enough GGTs to target the blood system of the prey or predator [37]. Nevertheless, GGT is an essential component of centipede survival, given the abundant expression of GGT. The molecular mechanism and candidate target of GGTs need to be further explored [38].

### 5.4. Other Enzymes

Others with less abundant enzymes were also reported in centipedes. Three glycosidic hydrolase families, chitinase, lysozyme and hyaluronidase, were found in centipede, while they did not exist in all centipede species. Chitinase may be helpful in the digestion of arthropods [85,86,87,88,89]. The lysozyme digests β-1,4-glycosidic bonds in the peptidoglycan of bacterial cell walls and can be used to kill bacteria [90,91]. Hyaluronidase is known as a “spreading factor” because it enhances the pathological effects of the venom components [79,92,93,94]. Glucose dehydrogenase initiates the catalytic process of the pentose phosphate pathway [95] and likely represents a case of the new functionalization of protein. Nonspecific esterases were reported in many taxa, including octopus [96], spiders [97,98], and snakes [99,100]. In centipedes, type B carboxyl esterase and homologous transcripts were discovered in many species except *E. rubripes* [37]. Similar to the type B carboxyl esterase, we do not know the role of *Porphyromonas*-type peptidyl arginine deiminase (PPAD), which was found in the *T. longicornis* venom [37]. Centipede PADs also existed in *Lithobius forficatus* by horizontal gene transfer from bacteria [19]. With the continuous upgrading of detection methods, the enzymes in centipede venom are continuously mined and identified. We will not enumerate them here.

### 5.5. Other Non-Enzymatic Proteins

The putative β pore-forming toxins (β-PFTx) are rich proteins in centipede venoms [37]. Upon proteolytic activation, the aerolysin-like β-PFTx is oligomerized into a pore-forming heptamer. CAPs (cysteine-rich proteins) exhibit multiple functions, including peptidases, vasodilators, myotoxins and ion channel modulators. CAP1-3 were reported from the *T. longicornis* and *E. rubripes*, Scolopendrinae and *S. morsitans* venoms [37]. In addition, the low-density lipoprotein receptor Class A repeat (LDLA) domain has been reported only in centipede, and no representative LDLAs from other venoms have been detected outside centipede [22,37], although its function remains to be determined.

## 6. Therapeutic Potential of Bioactive Peptides and Proteins from Centipede

Since time immemorial, humans have relied on naturally occurring substances for their therapeutic benefits. Venoms from multiple species encompassed a natural reserve of millions of active biological molecules. Thanks to their selectivity and stability, these molecules have proven invaluable tools for drug development, research, and discovery. For example, a series of bioactive peptides from the snakes, scorpions, spiders, honey bees, and cone snails venoms hold a promise as rich sources of chemotherapeutics against some human diseases, such as chronic inflammation, autoimmune disease, and cancer [101,102,103,104]. Over the years, venom-derived peptides and proteins have provided essential diagnostic and research tools [105,106,107,108,109]. Owing to their potency and precise targets, they provide insight into complex molecular interactions such as the coagulation cascade and are essential in drug discovery. Additionally, venom-derived molecular probes have greatly expounded our insight into the biophysical properties of different ion channel families, which is important in understanding the pharmacological properties and development of therapeutic agents for neurological, blood and other diseases (neurodegenerative diseases and brain ischemia).

Extensive research on the pharmacological properties is ongoing, and several of these venom-derived components are under clinical trials as potential therapeutic agents for several clinical indications. Currently, six FDA-approved venom-derived drugs are available on the market [110]. Pain is the most common presenting physical symptom and the primary reason for seeking medical care, which chronically affects people’s mental health and social life. As we know, Na_V_1.7 channels are promising analgesic targets for treating various pain-related diseases. The authors of this paper and other collaborators found a potent and selective inhibitor of the Na_V_1.7 channel, which exhibited a more potent analgesic than morphine in formalin-induced pain models. Thus, μ-SPTX-Ssm6a is a promising lead molecule for analgesic drug development. In addition, Na_V_ channels are well-established therapeutic targets, such as local anesthetics, antiarrhythmics, and anticonvulsants. Therefore, centipede venoms are potential therapeutics for related diseases. Likewise, K_V_ and Ca_V_ channels are potential therapeutic targets, including episodic ataxia, long-QT syndrome, epilepsy, benign familial neonatal convulsions, autosomal dominant non-syndromic hearing loss, hypokalemic periodic paralysis, night blindness, familial hemiplegic migraine and malignant hyperthermia [111]. Since the TRPV1 channel is widely distributed in the somatosensory system, the TRPV1 channel modulators, such as RhTx and RhTx2, can potentially treat pain and itch pathological conditions.

Microbial infections also present a significant global predicament, mainly due to the recalcitrance of pathogens to available prophylactic regimens. Therefore, there is an exigent need for new alternative-antibiotic therapies. AMPs derived from venoms have proven clinical efficacy in combating multidrug-resistant pathogens. Thus, the antimicrobial peptides from venomous animals, including centipedes, are lead candidate molecules for combating antibiotic-resistant bacteria. Following the study of the anticancer potential of centipede extracts, centipede venoms may aid the development of new anticancer agents.

**Figure 2:**
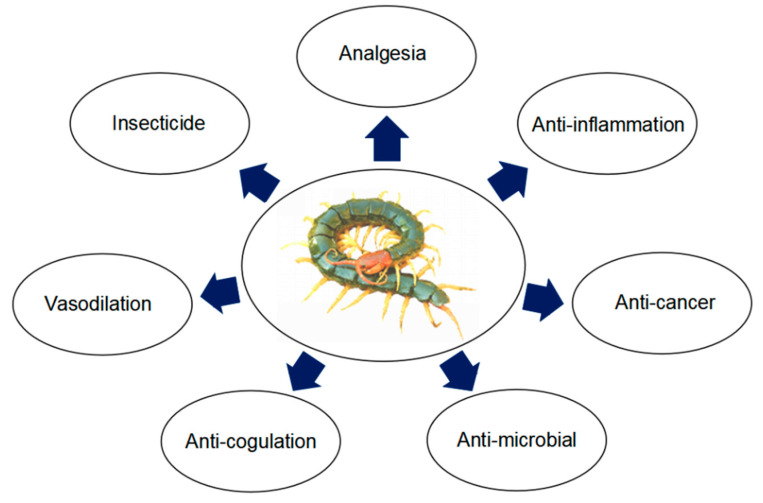
Functions of bioactive peptides and proteins from centipede.

Enzymes are abundant in centipede venom and have been proposed in various processes, including homeostatic balance, innate immunity, digestion, apoptosis and cell cycles [50,112,113,114,115,116]. Moreover, dysregulations or alterations of these enzymes lead to pathological conditions, such as rheumatic arthritis, osteoporosis and cardiovascular disorders [117,118]. Peptidase and peptidase inhibitors from centipede venom have therapeutic potential in immune-related diseases (Figure 2).

## 7. Conclusions

Animal venoms are abundant sources of bioactive peptides and proteins, which are used for the medical treatment of a broad range of diseases, including asthma, hypertension, cancer, cardiac failure, and polio [119,120,121]. Several venom-derived peptides from venomous animals have been clinically applied in cardiovascular, neurological disorders, and immune system diseases, such as captopril, ziconotide, tozuleristide, eptifibatide [119,122,123,124]. Despite the remarkable diversity of venomous animals, there appears to be a striking convergence regarding the types of proteins used in toxin scaffolds [37]. Nevertheless, our understanding of this fascinating area of evolution is limited by the small taxonomic range studied, with entire families of venomous animals almost entirely unknown. As an example, centipedes, class Chilopoda, may represent the oldest terrestrial venomous lineage after scorpions, having emerged approximately 440 Ma ago. Although the bioactive peptides and proteins from centipedes have not been fully appreciated and extensively studied, a prominent common attribute with most scorpion venoms, which is a well-studied group, is the presence of disulfide-rich peptides that act on the nervous system, cardiovascular system, musculoskeletal system and blood system. In addition, there are other differences in details. For instance, while both venoms contain neurotoxins that potently inhibit sodium channels, scorpion α-toxins specifically inhibit the fast inactivation mechanism of voltage-gated sodium channels leading to several neuro- and cardiotoxic effects.

With the development of an increasingly sensitive and accurate analytical method, more functional peptides and proteins from centipedes are being discovered. For instants, dozens of bioactive peptides containing two to four pairs of disulfide bonds have been cloned and isolated. Most of these peptidic components act on Na_V_, K_V_, and Ca_V_ channels, indicating that centipede venoms are good resources for discovering bioactive peptides and candidate-leading molecules. In addition, bioactive peptides and proteins are also excellent probes for exploring the structure and function of receptors and human physiology mechanisms. The investigation of centipede venom will promote people’s understanding of the components of centipede venom and provide lead molecules for the research and development of new drugs.

## Figures and Tables

**Table 1:** Representative functional components derived from centipede.

Venom Components	Centipede Species	Activities	Component Source	References
μ-SLPTX-Ssm1a	*S. subspinipes mutilans*	TTX-S Na_V_ channel inhibitor	Venom	[12]
μ-SLPTX-Ssm6a	*S. subspinipes mutilans*	Na_V_1.7 channel inhibitor	Venom	[21]
ω-SLPTX-Ssm1a	*S. subspinipes*	Activator of Cav channels in DRG	Venom	[12]
ω-SLPTX-Ssm2a	*S. subspinipes*	Inhibitor of Ca_V_ channels in DRG	Venom	[12]
κ-SLPTX-Ssm1a	*S. subspinipes mutilans*	Inhibitor of K_V_ channels in DRG	Venom	[12]
κ-SLPTX-Ssm2a	*S. subspinipes mutilans*	Inhibitor of K_V_ channels in DRG	Venom	[12]
κ-SLPTX-Ssm3a	*S. subspinipes mutilans*	Inhibitor of K_V_ channels in DRG	Venom	[12]
SSD559	*S. subspinipes dehaani*	Inhibitor of K_V_ channels in DRG	Venom	[22]
SsTx	*S. subspinipes mutilans*	Inhibitor of KCNQ4 and K_V_1.3 channel	Venom	[15]
SsTx-4	*S. subspinipes mutilans*	Inhibitor of Kir1.1, Kir4.1 and Kir6.2/SUR1 channels	Venom	[23]
SSD1052	*S. subspinipes dehaani*	Inhibitor of Ca_V_ channels in DRG	Venom	[22]
RhTx	*S. subspinipes mutilans*	TRPV1 channel activator	Venom	[24]
RhTx2	*S. subspinipes mutilans*	TRPV1 channel activator	Venom	[25]
Scolopendrin I	*S. subspinipes mutilans*	Antimicrobial activity	Venom	[26]
Scolopin 1	*S. subspinipes mutilans*	Antimicrobial activity	Venom	[27]
Scolopin 2	*S. subspinipes mutilans*	Antimicrobial activity	Venom	[27]
LBLP	*S. subspinipes mutilans*	Antifungal activity	Whole centipede	[28]
Scolopendin 1	*S. subspinipes mutilans*	Antimicrobial activity	Whole centipede	[29]
Scolopendin 2	*S. subspinipes mutilans*	Antimicrobial activity	Whole centipede	[30]
Scolopendrasin I	*S. subspinipes mutilans*	Antimicrobial activity	Whole centipede	[31]
Scolopendrasin II	*S. subspinipes mutilans*	Antimicrobial activity	Whole centipede	[32]
Scolopendrasin V	*S. subspinipes mutilans*	Antimicrobial activity	Whole centipede	[33]
Scolopendrasin VII	*S. subspinipes mutilans*	Antimicrobial activity; Anticancer activity	Whole centipede	[34]
Scolopendrasin IX	*S. subspinipes mutilans*	Anti-inflammatory activity	Whole centipede	[35]
TNGYT	*S. subspinipes mutilans*	FXa inhibitor	Venom	[36]
SSD14	*S. subspinipes dehaani*	γ-Glutamyl Transpeptidase, platelet aggregation and hemolytic activities	Venom	[22]
Trypsin-like S1 family	*S. subspinipes dehaani*	Serine peptidases, potentially involved in activation of toxins	Venom	[37,38]
Subtilisin-like S8 family	*E. rubripes* *C. westwoodi S. subspinipes dehaani*	Serine peptidases, potentially involved in activation of toxins	Venom	[37,38]
β-pore-forming toxins	Scolopendrids	In cell membranes, it has the potential to cause cytotoxicity by forming polymeric pores structures in cell membranes	Venom	[37,38]
CAP (cysteine rich proteins) protein	Scolopendrids	Unknown (CAP1, CAP3); Ca_V_ channel antagonist and trypsin inhibitor (CAP2)	Venom	[37,38]
Serotonin	*S. viridicornis*	Analgesic activity	Venom	[39,40]
Histamine	*S. subspinipes*	Analgesic activity	Venom	[41,42]
Transferrin	*E. rubripes* *S. morsitans*	Potential antimicrobial activity	Venom	[37,38]
Polysaccharide–protein complex	*S. subspinipes mutilans*	Inhibitor of tumor cells	Whole centipede	[43]
Hyaluronidase	Scolopendrids	Glycosaminoglycan degradation; potential for spreading of venom components	Venom	[37,44]
Cystatin type-1	*E. rubripes*	Potential peptidase inhibitors	Venom	[37,38]
Antithrombotic peptide SQL	*E. rubripes*	Inhibitor of platelet aggregation	Whole centipede	[45]
Lysozyme C	Scolopendrids	Potential antimicrobial activity	Venom	[37,38]
Scolonase	*S. subspinipes mutilans*	Fibrinolytic activity; Serine peptidase	Whole centipede	[46]
Phospholipase A2	*S. viridis* *S. subspinipes dehaan* *S. viridicornis* *O. pradoi*	Hydrolysis of glycerophospholipids; involved in anti-inflammatory, hemolysis, neurotoxicity, and cardiotoxicity	Venom	[15,47,48]
CentiPAD	*T. longicornis* *L. forficatus*	Peptidylarginine deiminase, potentially involved in post-translational modification of toxins	Venom	[19]
LDLA protein	Scolopendrids	Unknown	Venom	[37,38]

## Data Availability

No new data were created or analyzed in this study. Data sharing is not applicable to this article.
